# Which patients with heart failure should receive specialist palliative care?

**DOI:** 10.1002/ejhf.1240

**Published:** 2018-06-28

**Authors:** Ross T. Campbell, Mark C. Petrie, Colette E. Jackson, Pardeep S. Jhund, Ann Wright, Roy S. Gardner, Piotr Sonecki, Andrea Pozzi, Paula McSkimming, Alex McConnachie, Fiona Finlay, Patricia Davidson, Martin A. Denvir, Miriam J. Johnson, Karen J. Hogg, John J.V. McMurray

**Affiliations:** ^1^ BHF Cardiovascular Research Centre University of Glasgow Glasgow UK; ^2^ SNAHFS Golden Jubilee National Hospital Scotland UK; ^3^ Queen Elizabeth University Hospital Scotland UK; ^4^ Hospital Papa Giovanni XXIII Bergamo Italy; ^5^ Robertson Centre for Biostatistics University of Glasgow Glasgow UK; ^6^ Johns Hopkins University Baltimore MD USA; ^7^ Edinburgh University Edinburgh UK; ^8^ Hull York Medical School University of Hull Hull UK; ^9^ Glasgow Royal Infirmary Glasgow UK

**Keywords:** Heart failure, Palliative care

## Abstract

**Aims:**

We investigated which patients with heart failure (HF) should receive specialist palliative care (SPC) by first creating a definition of need for SPC in patients hospitalised with HF using patient‐reported outcome measures (PROMs) and then testing this definition using the outcome of days alive and out of hospital (DAOH). We also evaluated which baseline variables predicted need for SPC and whether those with this need received SPC.

**Methods and results:**

PROMs assessing quality of life (QoL), symptoms, and mood were administered at baseline and every 4 months. SPC need was defined as persistently severe impairment of any PROM without improvement (or severe impairment immediately preceding death). We then tested whether need for SPC, so defined, was reflected in DAOH, a measure which combines length of stay, days of hospital re‐admission, and days lost due to death. Of 272 patients recruited, 74 (27%) met the definition of SPC needs. These patients lived one third fewer DAOH than those without SPC need (and less than a quarter of QoL‐adjusted DAOH). A Kansas City Cardiomyopathy Questionnaire (KCCQ) summary score of <29 identified patients who subsequently had SPC needs (area under receiver operating characteristic curve 0.78). Twenty‐four per cent of patients with SPC needs actually received SPC (*n* = 18).

**Conclusions:**

A quarter of patients hospitalised with HF had a need for SPC and were identified by a low KCCQ score on admission. Those with SPC need spent many fewer DAOH and their DAOH were of significantly worse quality. Very few patients with SPC needs accessed SPC services.

## Background

Patients with heart failure (HF) have a major symptom burden, considerable impairment of quality of life (QoL) and high rates of admission, re‐admission and death.^1^, ^2^ Palliative care (PC) is defined by the World Health Organisation (WHO) as an approach that improves the QoL of patients and their families facing a life‐threatening illness.^3^ It seems intuitive that some or even many patients with HF would benefit from PC and several guidelines advocate use of PC, alongside usual care, in selected patients with HF.^4^, ^5^ A recent policy statement goes further, recommending that PC is integrated into the routine care of all patients with advanced HF, with most needs managed by the usual care team.^6^ Those with more challenging PC needs should have access to specialist PC (SPC) providers working in collaboration with the usual‐care team.^6^ While PC is a treatment which, in principle, can be delivered by all health care professionals, SPC is provided by multi‐professional team who have undergone specialist training in PC. SPC services often include access to additional resources such as inpatient or outpatient hospice care. However, which patients should be selected is not clear and most clinicians do not find it easy to identify those who should be referred to SPC services.^7^ This issue has been highlighted as requiring further research in the European Society of Cardiology (ESC) Heart Failure Association position statement on PC use in HF.^8^ Existing studies on this subject have many limitations. For example, most enrolled highly selected cohorts, did not follow patients over time (i.e. used one‐off assessments), or did not fully characterise the patients studied (for example by recording the severity of HF or treatment received).^9^

Therefore, our goal was to develop and to test a simple and practical definition of who needs SPC in a cohort of near‐consecutive patients admitted to hospital with worsening HF. We also compared patients who actually received PC with those in need of SPC, according to our definition.

## Methods

### Study patients and protocol

The design and rationale of this study are published.^10^ In a single centre serving as a community hospital, near‐consecutive patients with suspected HF were screened for inclusion in the study between 9 January 2013 and 1 December 2014 (near‐consecutive means that patients were recruited consecutively except when the single recruiting physician‐investigator was on vacation). The ESC guidelines were used to define HF.^11^ Patients were eligible for inclusion if they had signs and symptoms of HF, a B‐type natriuretic peptide (BNP) concentration > 100 pg/mL, and objective evidence of heart disease or dysfunction on echocardiography (either left ventricular systolic dysfunction, elevated left ventricular filling pressures, or significant valve disease). Patients were excluded if they were unable or unwilling to provide written informed consent.

During the index admission, medical and drug history, physical examination, laboratory, and echocardiographic results were recorded. A physician assessment of performance status was made using the Australia modified Karnofsky Performance Status (AKPS) score, an ‘end‐of‐the‐bed’ assessment ranging between 0–100, with lower scores indicating lower performance status.^12^ A HF‐specific assessment of patients' needs, using the Needs Assessment Tool‐Progressive Disease‐Heart Failure (NAT‐PD‐HF), was also completed during index admission,^13^ with patients deemed to have important needs if they were assessed as having ‘significant concern’ on any of the NAT‐PD‐HF patient wellbeing domains. Caregiver burden was assessed using the Zarit Burden Interview (ZBI) questionnaire.^14^

### Patient‐reported outcome measures

Patients completed a variety of patient‐reported outcome measures (PROMs) chosen to quantify different potential PC needs including QoL, symptom burden, and mood disturbance. Disease‐related QoL was assessed by the Kansas City Cardiomyopathy Questionnaire (KCCQ) and general QoL using the Short Form 12 (SF‐12) questionnaire.^15^, ^16^ Symptom burden was assessed by the Edmonton Symptom Assessment Scale (ESAS).^17^ Mood disturbance was assessed using the Hospital Anxiety and Depression Scale (HADS).^18^ At every study assessment all PROMs were repeated. PROMs were categorised according to severity. The derivation and testing of these PROMs in HF (as well as definitions of severity where available) are described in *Table* 1.^15^, ^16^, ^18^, ^19^, ^20^, ^21^

**Table 1:** Patient‐reported outcome measure severity cut‐off definition

**PROM**	**Severity**	**Cut‐off**	**Severity cut‐off derivation**
HADS Depression	None/mild	≤10	Severity cut‐off scores suggested by authors, corroborated by normative data.^18^, ^20^
	Moderate	11–15
	Severe	≥16
HADS Anxiety	None/mild	≤10	Severity cut‐off scores suggested by authors, corroborated by normative data.^18^, ^20^
	Moderate	11–15
	Severe	≥16
KCCQ summary score	None/mild	>50	Correlation of cut‐offs with NYHA class, mortality, and PC needs.^15^, ^19^
	Moderate	25–50
	Severe	<25
ESAS summary score	None/mild	0–33	Individual symptom score severity based on HF cohort.^21^ No summary score severity available.
	Moderate	34–66
	Severe	67–100
SF‐12 Physical summary score	None/mild	>40.28	Severity scores based on normative data: moderate = between 2–3 SD from mean; severe = >3 SD from mean.^16^
	Moderate	30.56–40.28
	Severe	<30.56
SF‐12 Mental summary score	None/mild	>40.28	Severity scores based on normative data: moderate = between 2‐3 SD from mean; severe = >3 SD from mean.^16^
	Moderate	30.56–40.28
	Severe	<30.56
Overall severity category	Severely impaired	Severe in any PROM category	

ESAS, Edmonton Symptom Assessment Scale; HADS, Hospital Anxiety and Depression Scale; HF, heart failure; KCCQ, Kansas City Cardiomyopathy Questionnaire; NYHA, New York Heart Association; PC, palliative care; PROM, patient‐reported outcome measure; SD, standard deviation; SF‐12, Short Form 12.

Patients were reviewed every 4 months (for a minimum of 8 and up to a maximum of 28 months). Study visits took place at the study centre or in the patients' own home, if they were too frail to attend the study centre, or expressed a preference for this. Patients were also followed up for a minimum of 12 months and up to a maximum 21 months using electronic medical record linkage to document re‐admission and death. As described previously, the Scottish National Health Service electronic medical record linkage enables follow‐up for death (including location) and hospitalisation (including cause).^22^ Electronic patient records, PC registries, and hospice records were searched to identify participants who accessed PC services.

The study team were not involved, nor did they influence the care of patients. Patients admitted to hospital due to HF were treated as per current guidelines.^4^ In our area usual practice is for patients to attend follow‐up clinic appointments with either a cardiologist or general physician, as well as a general practitioner, and a HF liaison nurse. The specific care patients received following discharge was not recorded as part of this study.

The study protocol was approved by the local ethics committee and the study was conducted according to the principles of the Declaration of Helsinki. All participants provided written, informed consent. We attest that we have obtained appropriate permissions and paid any required fees for use of copyright protected materials.

### Definition of need for specialist palliative care

**Figure 1:**
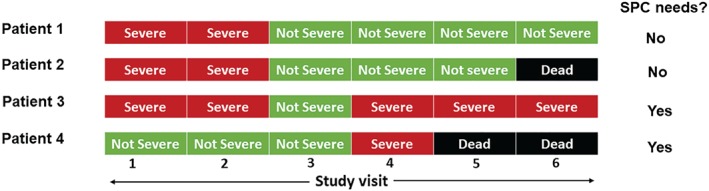
Definition of specialist palliative care (SPC) needs: persistent impairment (≥2 consecutive assessments) of any patient‐reported outcome measure (PROM) without recovery, or severe impairment of any PROM without recovery preceding death. Patient 1 did not have SPC needs as there was improvement of PROM(s) whereas there was persistent impairment without improvement in patient 3. Patient 4 has SPC needs as there was severe impairment of PROM(s) preceding death, whereas patient 2 did not have severe impairment preceding death.

We defined need for SPC as severe impairment of any PROM persisting for two or more consecutive study visits without improvement, or severe impairment of any PROM followed by death before the PROM was repeated, i.e. no improvement was reported in the PROM before death (*Figure* 1). Patients who missed a study assessment were assumed to have the same PROM score as previously recorded, that is, we used last observation carried forward. There was no limit on how far forward observations were carried.

### Testing of definition of need for specialist palliative care against days alive and out of hospital (DAOH) and quality of life‐adjusted DAOH

Our definition of need for SPC was tested against DAOH, QoL‐adjusted, symptom‐adjusted, and mood‐adjusted DAOH. DAOH is a measure that takes account of the length of the index hospitalisation, days of re‐hospitalisation, and days of life lost due to death. For example, a patient followed for a fixed period of 180 days with 10 days of index hospitalisation, two further admissions of 10‐ and 15‐day duration, respectively, followed by death at day 135 would have 100 DAOH (out of a possible 180). To calculate QoL‐adjusted DAOH, the number of DAOH between each study assessment was calculated (online supplementary *Figure* S1) and these days were then adjusted according to the KCCQ overall summary score, to quality‐weight each DAOH. A higher KCCQ score equates to better QoL and lower score to poorer QoL, with a range of 0–100. For example, if a patient spent 100 DAOH and had a KCCQ summary score of 75 consistently over that period, this would be calculated as 100 x 0.75 QoL‐adjusted DAOH, equating to 75 days of good health spent out of hospital, or 25 of 100 days lost due to poor QoL. The proportion of QoL‐adjusted DAOH compared with the potential DAOH (all alive and in full health, and without hospital admission) during the whole study was calculated for each patient. No patient was excluded from this analysis, as missing KCCQ scores were carried forward from the previous study assessment. This analysis was then repeated, adjusting DAOH for symptom burden using the ESAS summary score, HADS summary score, and both the physical and mental summary QoL scores of the SF‐12, instead of KCCQ.

### Statistical analysis

Continuous variables are expressed as median (interquartile range), unless otherwise specified. Comparisons of categorical variables were performed using Fisher's exact test, and continuous variables were compared using the Mann–Whitney U test. Multivariable logistic regression was used to calculate odds ratios and 95% confidence intervals for baseline variables associated with the need for SPC. Baseline variables tested included common markers of prognosis,^23^ performance status assessment (using the AKPS),^12^ needs assessment (using the NAT‐PD‐HF), and PROM summary scores. Cut‐off scores and area under the receiver operating characteristic curve for continuous variables identified as predictors of SPC needs were calculated using the Youden method.^24^ All statistical analyses were performed using SAS® v9.2 (SAS Institute Inc., Cary, NC, USA).

## Results

A total of 829 near‐consecutive patients with suspected HF were screened for inclusion between 9 January 2013 and 1 December 2014. Of these, 272 met the inclusion criteria and agreed to participate in the study (online supplementary *Figure* S2). The median time from admission to baseline assessment was 2 (Q1‐Q3: 2–4) days. Median length of stay was 9 (5–15) days. Median follow‐up via record‐linkage was 775 (608–913) days. There were 103 (38%) deaths during follow‐up, four during the index hospital admission. Overall, 217 (80%) patients were re‐admitted to hospital during follow‐up (for any reason). In total, 963 individual patient assessments were completed. Thirty‐eight per cent of all study visits were carried out in patients' homes. A detailed description of the proportion of patients attending each study assessment, including the number of patients with missing data for each PROM at each time point, is provided in the online supplementary *Table* S1. The proportion of patients with missing PROM data at each study assessment for reasons other than death, increased over time, from 19% at month 4 to 33% at month 24. No patients were lost to follow‐up for vital status.

### PROMs at baseline

The findings for the individual PROMs at baseline are detailed in *Table* 2. During the index hospitalisation, 114 (42%) patients had severe impairment of at least one PROM (online supplementary *Table* S2). More patients had a severely reduced KCCQ than a severe score for any other PROM. Of the 114 patients who scored severe in any PROM at baseline, 55 (48%) did so in two or more PROMs. Patients in the group defined as needing SPC (see below) had worse median summary scores and a higher proportion had a severely impaired score for each PROM.

**Table 2:** Baseline patient‐reported outcome measure results

	**SPC needs (*n* = 74)**	**Not SPC needs (*n* = 198)**	***P*‐value**
**QoL assessment**			
KCCQ severity, *n* (%)			<0.001
None/mild	6 (8)	54 (27)	
Moderate	17 (23)	100 (51)	
Severe	51 (69)	44 (22)	
KCCQ summary score	16 (8–27)	39 (27–52)	<0.001
SF‐12 Physical severity, *n* (%)			<0.001
None/mild	22 (31)	101 (57)	
Moderate	34 (48)	57 (32)	
Severe	15 (21)	20 (11)	
SF‐12 Physical summary score	27 (21–31)	32 (25–38)	<0.001
SF‐12 Mental severity, *n* (%)			<0.001
None/mild	45 (63)	154 (87)	
Moderate	15 (21)	22 (12)	
Severe	11 (16)	2 (1)	
SF‐12 Mental summary score	36 (26–44)	45 (37–53)	<0.001
**Symptom burden assessment**			
ESAS severity, *n* (%)			<0.001
None/mild	14 (19)	94 (48)	
Moderate	45 (62)	90 (46)	
Severe	14 (19)	12 (6)	
ESAS summary score	53 (36–63)	34 (18–49)	<0.001
**Mood assessment**			
HADS Anxiety severity, *n* (%)			<0.001
None/mild	38 (54)	158 (82)	
Moderate	22 (31)	28 (15)	
Severe	11 (16)	6 (3)	
HADS Anxiety summary score	10 (6–13)	6 (3–9)	<0.001
HADS Depression severity, *n* (%)			<0.001
None/mild	44 (61)	174 (89)	
Moderate	24 (33)	15 (8)	
Severe	4 (6)	6 (3)	
HADS Depression summary score	9 (7–12)	6 (3–9)	<0.001

Values are expressed as median (interquartile range) unless otherwise stated.

ESAS, Edmonton Symptom Assessment Scale; HADS, Hospital Anxiety and Depression Scale; KCCQ, Kansas City Cardiomyopathy Questionnaire; SPC, specialist palliative care; QoL, quality of life; SF‐12, Short Form 12.

**Figure 2:**
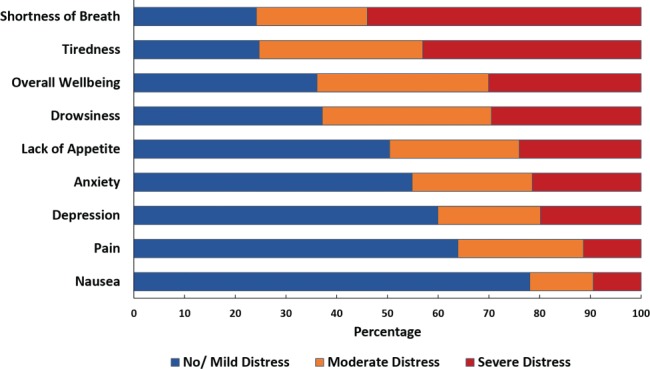
Edmonton Symptom Assessment Scale: symptom distribution at baseline.

**Figure 3:**
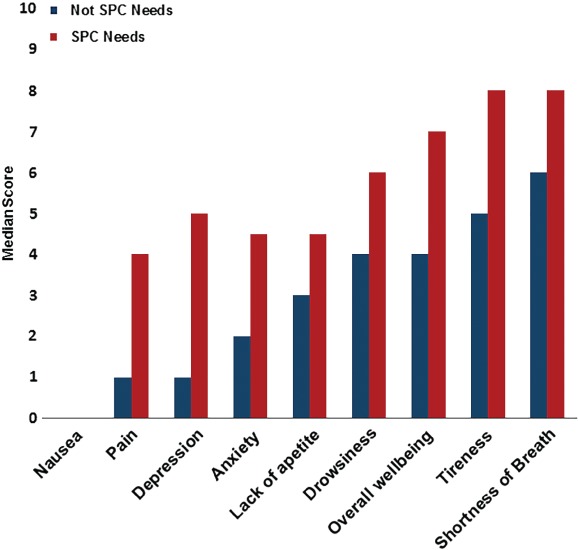
Median symptom score in patients with and without specialist palliative care (SPC) needs.

Symptom burden, assessed using the ESAS scale, was high during the index admission (*Figure* 2), and patients with SPC needs reported higher burden for each individual symptom, except nausea (*Figure* 3).

### PROMs during follow‐up

The proportion of patients at each study assessment who scored severe for each PROM, and the number of PROMs with a severe scoring are shown in the online supplementary *Table* S2. Compared with baseline, the percentage of patients who were classified as severe for any PROM reduced during follow‐up, but ranged between 26% and 36%. Of these patients, most had severe impairment of disease‐related QoL as assessed by the KCCQ, followed by impairment of general QoL as assessed by the SF‐12 Physical, and then symptom burden as assessed by the ESAS.

### Prevalence of specialist palliative care needs

Of the 272 patients in this study, 74 (27%) had SPC needs using the definition described in the Methods, i.e. persistently (≥2 consecutive study visits) severe impairment of any PROM without improvement, or severe impairment of any PROM followed by death before further PROMs could be recorded. Of the 74 patients with SPC needs, 47 (64%) met our definition by having severe impairment of at least one PROM preceding death and 46 (62%) qualified by having persistently severe impairment of any PROM without improvement (20 of 46 of the latter patients died during follow‐up).

### Clinical characteristics of patients with a need for specialist palliative care

The clinical characteristics of those with and without a need for SPC are detailed in *Table* 3. Patients with a need for SPC had a worse New York Heart Association (NYHA) class distribution prior to admission, and a higher proportion of patients had been hospitalised in the preceding 6 months for worsening HF, compared to those without SPC needs. Physician assessed performance status (using the AKPS) was lower (i.e. worse) in patients with SPC needs and a higher proportion of those with SPC needs were classified by a physician as having significant needs using the NAT‐PD‐HF. Of the caregivers interviewed, 93 (34%) were available and/or willing to complete the ZBI; for patients with SPC needs, caregivers recorded worse overall scores and a higher proportion of caregivers reported a moderate‐to‐severe burden in the group defined as having a need for SPC, compared to those without such a need.

**Table 3:** Baseline clinical characteristics

	**SPC needs (*n* = 74)**	**Not SPC needs (*n* = 198)**	***P*‐value**
Age, years	73 (65–81)	77 (71–83)	**0.041**
Female sex	28 (38)	100 (51)	0.076
SBP, mmHg	127 (112–152)	136 (120–158)	**0.018**
BMI, kg/m^2^	28 (24–32)	26 (24–32)	0.245
NYHA class			**0.031**
II	20 (27)	62 (31)	
III	33 (45)	108 (55)	
IV	21 (28)	28 (14)	
Medical history			
Previous HF diagnosis	38 (51)	82 (41)	0.170
HF diagnosis >2 years	19 (26)	58 (29)	0.651
HF hospitalisation in preceding 6 months	10 (14)	12 (6)	0.077
Hypertension	52 (70)	132 (67)	0.663
Myocardial infarction	41 (55)	70 (35)	**0.004**
Atrial fibrillation	41 (55)	103 (52)	0.683
TIA/CVA	11 (15)	41 (21)	0.385
Peripheral arterial disease	11 (15)	27 (14)	1.000
Diabetes	32 (43)	57 (29)	**0.029**
COPD	23 (31)	46 (23)	0.211
Depression	14 (19)	23 (12)	0.164
Cancer	7 (10)	24 (12)	0.670
ICD/CRT	5 (7)	13 (7)	1.000
Discharge medication			
ACEi/ARB	48 (65)	137 (69)	0.559
Beta‐blocker	48 (65)	144 (73)	0.232
MRA	24 (32)	67 (34)	0.886
Digoxin	20 (27)	56 (28)	0.880
Laboratory			
BNP, pg/mL	807 (471–1810)	683 (417–1329)	0.192
Na^+^, mmol/L	138 (134–140)	138 (135–140)	0.621
eGFR, mL/min/1.73 m^2^	55 (38–80)	63 (40–82)	0.639
Hb, g/L	120 (108–133)	123 (109–138)	0.346
Echocardiography			
EF ≤50%	52 (70)	131 (66)	0.564
EF, %	36 (24–50)	40 (27–54)	0.191
LVIDD/BSA, mm/m^2^	30 (26–35)	29 (26–33)	0.397
Physician completed assessments			
AKPS	60 (50–80)	80 (60–90)	**<0.001**
NAT‐PD‐HF significant concern	28 (38)	42 (21)	**0.008**
Caregiver burden assessment			
ZBI severity*			**<0.001**
None/mild	9 (33)	48 (73)	
Mild–moderate	12 (44)	13 (20)	
Moderate–severe	6 (22)	5 (8)	
ZBI summary score	24 (15–38)	12 (6–22)	**<0.001**

Values are expressed as n (%) or median (interquartile range).

ACEi, angiotensin‐converting enzyme inhibitor; ARB, angiotensin receptor blocker; AKPS, Australia modified Karnofsky Performance Status; BMI, body mass index; BSA, body surface area; BNP, B‐type natriuretic peptide; COPD, chronic obstructive pulmonary disease; CRT, cardiac resynchronisation therapy; CVA, cerebral vascular accident; EF, ejection fraction; eGFR, estimated glomerular filtration rate; Hb, haemoglobin; HF, heart failure; ICD, implantable cardioverter defibrillator; LVIDD, left ventricular internal diameter in diastole; MRA, mineralocorticoid receptor antagonist; Na^+^, sodium; NAT‐PD‐HF, Needs Assessment Tool‐Progressive Disease‐Heart Failure; NYHA, New York Heart Association; SBP, systolic blood pressure; SPC, specialist palliative care; TIA, transient ischaemic attack; ZBI, Zarit Burden Interview.

* 93 caregivers completed the ZBI questionnaire at baseline.

A greater proportion of those who developed SPC needs were men, although this difference was not statistically significant (*P* = 0.076). Patients who developed SPC needs were also younger (*P* = 0.041) and had a more frequent history of myocardial infarction (*P* = 0.004) and diabetes (*P* = 0.029), but did not have more total co‐morbidity overall and did not have a significantly higher BNP or lower estimated glomerular filtration rate (eGFR) or haemoglobin (but did have a lower systolic blood pressure, *P* = 0.018). A longer standing diagnosis of HF (>2 years) was no more common in patients with a need for SPC, compared to those without.

### Testing the definition of need for specialist palliative care–days spent alive and out of hospital

Patients meeting our definition of needing SPC spent one third fewer DAOH (not adjusted for QoL) than those without a need for SPC; specifically, the median number of DAOH in patients with a need for SPC was 402 (171–598) compared with 635 (419–802) for those not meeting the definition of needing SPC (*P* < 0.001).

**Figure 4:**
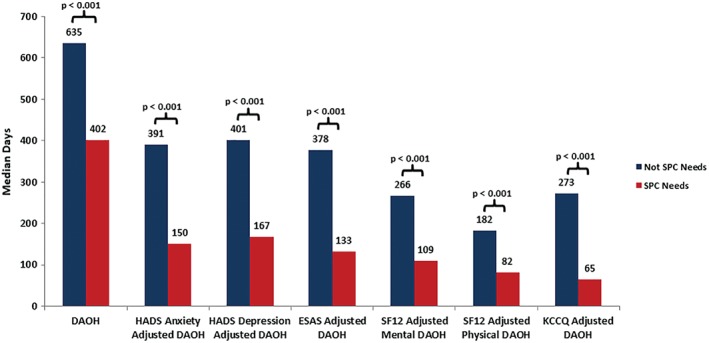
Quality of life‐adjusted, symptom‐adjusted, and mood‐adjusted days alive and out of hospital (DAOH) analysis. ESAS, Edmonton Symptom Assessment Scale; HADS, Hospital Anxiety and Depression Scale; KCCQ, Kansas City Cardiomyopathy Questionnaire; SF12, Short Form 12; SPC, specialist palliative care.

After adjusting each DAOH for symptom burden using the ESAS, patients with SPC needs had under half the number of symptom‐adjusted DAOH as those who did not meet the definition of SPC needs (*Figure* 4). Patients with SPC needs had a similar reduction in QoL and mood‐adjusted DAOH, using the mental and physical components of the SF‐12 and HADS, respectively. After adjusting each DAOH for impairment of QoL using the KCCQ, patients with SPC needs enjoyed less than one quarter of the number of QoL‐adjusted DAOH of those without a need for SPC.

### Prediction of which patients need specialist palliative care

Results of the multivariable analysis of baseline prognostic markers using the MAGGIC (Meta‐Analysis Global Group in Chronic Heart Failure) risk model,^25^ performance status assessment, needs assessment and PROMs are detailed in *Table* 4. The strongest predictor of developing a need for SPC was a low KCCQ score. The optimal KCCQ cut‐off for this purpose was a score of <29, giving a sensitivity and specificity of 80% and 71%, respectively, and an area under the receiver operating characteristic curve of 0.78. As the KCCQ was the PROM which was most commonly severely impaired in our cohort (i.e. identifying a need for PC by our definition), a sensitivity analysis was performed by omitting KCCQ from the definition of PC needs (i.e. only using a persistently severe impairment of the other PROMs/a severe impairment followed by death before repeat assessment of the PROM). This resulted in 49 (18%) of patients meeting our definition of a need for PC. In this sensitivity analysis using the full multivariable model, KCCQ <29 remained the strongest and only significant predictor of SPC (online supplementary *Table* S3).

**Table 4:** Multivariable logistic regression of predictors of specialist palliative care needs

**Variable**	**OR (95% CI)**	***P*‐value**
MAGGIC risk score, per 5 unit increase	1.03 (0.76–1.40)	0.861
AKPS score, per 10 unit increase	0.98 (0.95–1.01)	0.161
NAT‐PD‐HF significant needs	1.03 (0.44–2.42)	0.947
KCCQ summary score, per unit increase	0.97 (0.94–0.99)	**0.021**
HADS Depression summary score, per unit increase	1.03 (0.92–1.15)	0.610
HADS Anxiety summary score, per unit increase	1.03 (0.94–1.13)	0.526
ESAS summary score, per unit increase	1.00 (0.98–1.02)	0.952
SF‐12 Physical summary score, per unit increase	0.97 (0.92–1.03)	0.311
SF‐12 Mental summary score, per unit increase	0.97 (0.92–1.02)	0.222

AKPS, Australia modified Karnofsky Performance Status; CI, confidence interval; ESAS, Edmonton Symptom Assessment Scale; HADS, Hospital Anxiety and Depression Scale; KCCQ, Kansas City Cardiomyopathy Questionnaire; MAGGIC, Meta‐Analysis Global Group in Chronic Heart Failure; NAT‐PD‐HF, Needs Assessment Tool‐Progressive Disease‐Heart Failure; OR, odds ratio; SF‐12, Short Form 12.

### Use of specialist palliative care services

Of the 272 patients studied, 32 (12%) accessed SPC services, either as an inpatient or outpatient during follow‐up. Of the 74 patients who met our definition of SPC needs, 18 (24%) received SPC, compared to 14 (7%) of the 198 patients who did not meet the definition of SPC needs (*P* < 0.001). Five patients (7%) with a need for SPC received hospice care, compared with one (0.5%) of those without a need for SPC.

## Discussion

Although it can be argued that all patients with HF should receive PC, in that improvement in QoL is a treatment goal for everyone, not all patients require SPC input. Physicians find it difficult to identify which patients should be referred for SPC. We have proposed and tested a definition of who will develop a need for SPC and shown how this need can be identified before discharge in patients hospitalised with worsening HF. Approximately one quarter of a cohort of 272 nearly consecutively recruited patients admitted to hospital with HF met this definition. Patients fulfilling our definition had a greatly reduced number of DAOH (one third fewer days than those without a need for SPC) and an even more striking reduction in disease‐related QoL‐adjusted DAOH (one quarter of those without a need for SPC). In investigating the need for SPC, we used a broader range of quantifiable PROMs than in any previous study and investigated a ‘real world’ group of patients. We found that from among the PROMs used, the KCCQ performed best and a score < 29 (out of 100) during admission was the strongest predictor of PC needs during follow‐up. By recruiting a near‐consecutive cohort, we believe that our results can be generalised to patients seen every day in clinical practice.

Overall, the patients in our study were elderly, had multiple co‐morbidities, and most had a reduced ejection fraction, in keeping with epidemiological studies and registries of patients hospitalised with HF.^26^, ^27^ Our definition of need for SPC identified a subset among these patients with characteristics that intuitively make sense: they had worse prior NYHA class, were more likely to have been recently hospitalised with HF and had other, independent, measures of poor performance (AKPS score) and needs (using the NAT‐PD‐HF), as well as corroborating evidence of greater caregiver burden (ZBI). Importantly, these patients were not clearly identified by traditional physiological markers of advanced HF such as low eGFR, or conventional prognostic markers such as ejection fraction or BNP.

Patients with a need for SPC received similar treatment for HF, compared to those without. This is an important additional finding as most prior studies looking at potential SPC need in patients with HF did not describe use of conventional HF therapy. SPC, as an additional intervention, should mainly be considered in patients who continue to have a high symptom burden and poor QoL despite use of evidence‐based HF therapy.^24^ Prescription of disease‐modifying pharmacotherapy was high in our study, including in patients with a need for SPC, although very few patients had a device (e.g. implantable cardioverter defibrillators, cardiac resynchronisation therapy, ventricular assist devices) implanted.

We believe that our definition is intuitive and identifies patients who have either poor QoL combined with greatly reduced life expectancy or sustained and substantial impairment of QoL. It accounts for the fluctuating nature of HF by using longitudinal assessments. By using PROMs, the patient's perspective of the influence HF has on their lives is described, rather than taking the physician's perspective.

We have shown that the KCCQ summary score, assessed during a hospital admission, can identify patients who had or go on to develop a need for SPC. This measure was a more powerful predictor of SPC need than variables usually considered predictors of prognosis or variables reflecting performance status. Our analysis shows that a KCCQ summary score of <29 is the best tool, among a variety of PROMs, to identify those with a need for SPC and this simple and reproducible PROM could easily be adopted into clinical practice. One other study has also suggested that the KCCQ might be useful for this purpose. In a retrospective examination of the EVEREST (Efficacy of Vasopressin Antagonism in Heart Failure Outcome Study with Tolvaptan) clinical trial, need for SPC was arbitrarily defined as a KCCQ score persistently <45 or death within 6 months. In a multivariable analysis, a KCCQ score of <25 was the strongest predictor of need for SPC defined in this way.^19^ Although our study and the EVEREST analysis are complementary, their design was quite different in that we enrolled a near‐consecutive and unselected cohort of patients with HF, tested a variety of PROMs, conducted more frequent patient assessments and tested our definition of need for SPC using a different outcome. Nevertheless, the KCCQ emerged as the best PROM for predicting those with a need for SPC. However, using a PROM for this purpose is not possible for all patients admitted to hospital with HF, as not every patient can complete a PROM, such as those with cognitive impairment, or those with visual impairment or reading difficulties.

The ability to identify patients with SPC needs is potentially valuable from several perspectives. These individuals could be the focus of efforts to improve QoL, attempts to prevent re‐admissions or interventions to improve quality of death, such as those suggested in the ESC Heart Failure Association PC position statement.^8^ A KCCQ score of <29 could also be used as the main inclusion criterion for clinical trials aimed at testing SPC interventions.

Despite over one quarter of patients meeting the definition of need for SPC, very few patients accessed either SPC services or hospice care; indeed, 74% of those with a SPC need did not access these services. Perhaps because of uncertain criteria for who to refer, few patients with HF receive SPC. Of all patients accessing hospice care or SPC services in England and Wales in 2013–14, 88% had a diagnosis of cancer.^28^ In a recent audit of 54 654 patients admitted to hospital with HF in England and Wales between 2013–14, only 4% were referred to SPC during admission.^29^ Two recent studies in the United States retrospectively analysed large HF databases to identify patients thought likely to have SPC needs.^30^, ^31^ These studies identified 4474 and 32 270 patients, respectively, thought to have SPC needs, of whom 7.6% and 9.6% were seen by SPC services.

### Study limitations

This study was conducted in a single secondary care centre which potentially reduces the generalisability of the results. However, this issue is mitigated by the benefit gained from reduction in selection bias by recruiting a prospective, near‐consecutive, and unselected cohort. Another potential weakness of the study was missed study assessments. These were unavoidable in a longitudinal study assessing an elderly and co‐morbid population. However, overall there were high study retention rates, due to the high proportion of study assessments taking place in patients' homes. No patients were lost to follow‐up for vital status. The overall proportion of missing PROM data, for reasons other than death, was low, although we have made an assumption that patients had the same PROM score at subsequent missed assessments. This represents a potential weakness given the fluctuant course of HF.

When calculating QoL‐, mood‐, and symptom‐adjusted DAOH, we have made an assumption that the patient had the same KCCQ, SF‐12, HADS, and ESAS score for each day until a subsequent assessment was performed, i.e. last observation carried forward. Although these PROMs were not designed or validated to provide an assessment over 4 months, we chose to use this frequency of assessment to minimize patient study fatigue and to encourage study retention.

To our knowledge there are no validated cut points for ‘mild’, ‘moderate’ and ‘severe’ grades of the ESAS summary score. Arbitrary points were therefore used, but this makes an assumption that each symptom has the same weight in its effect on QoL. However, although this may not be the case, the findings are consistent with the other measures of need.

Fourteen patients (7%) who did not meet our definition of SPC need did access SPC services. This could potentially reflect a weakness in the sensitivity of our definition of SPC need. However, the individual reasons for referral to SPC were not available, and given the co‐morbid and elderly study patient population, it is possible that some of these (and those with HF SPC needs) accessed SPC services for another pathology such as cancer for which the referral systems are much more robust.

## Conclusion

We proposed and tested a definition in hospitalised HF patients who subsequently need SPC; 27% of patients met this definition. Patients who met this definition enjoyed only about a quarter of the QoL‐adjusted DAOH of those who did not. A KCCQ score of <29 on admission identified patients who go on to have SPC needs after discharge.

### Funding

This study was funded by a project grant from the British Heart Foundation; grant number PG/13/17/30050.

**Conflict of interest:** none declared.

## Supporting information


**Table S1.** Completion of patient‐reported outcome measures.
**Table S2.** Severity of PROM by study visit.
**Table S3.** Multivariable logistic regression of predictors of palliative care needs – sensitivity analysis –KCCQ removed from definition of palliative care needs.
**Table S4.** Multivariable logistic regression of predictors of palliative care needs best fit model using backwards selection.
**Table S5.** Multivariable logistic regression of predictors of palliative care needs best fit model using backwards selection – sensitivity analysis – KCCQ removed from definition of palliative care needs.
**Figure S1.** Calculation of days alive and out of hospital.
**Figure S2.** Screening and recruitment.
