# Supplementary material for: Which patients with heart failure should receive specialist palliative care?
Source: Eur J Heart Fail. 2018 Jun 28;20(9):1338–47. doi: 10.1002/ejhf.1240 (PMC6607479; doi:10.1002/ejhf.1240)
Supplement: Supplementary file 1 — Table S1. Completion of patient‐reported outcome measures. Table S2. Severity of PROM by study visit. Table S3. Multivariable logistic regression of predictors of palliative care needs – sensitivity analysis –KCCQ removed from definition of palliative care needs. Table S4. Multivariable logistic regression of predictors of palliative care needs best fit model using backwards selection. Table S5. Multivariable logistic regression of predictors of palliative care needs best fit model using backwards selection – sensitivity analysis – KCCQ removed from definition of palliative care needs. Figure S1. Calculation of days alive and out of hospital. Figure S2. Screening and recruitment. [file EJHF-20-1338-s001.docx]

# **SUPPLEMENTARY MATERIAL**

**Supplementary Tables**

**Table S1** Completion of patient reported outcome measures

**Table S2** Severity of PROM by study visit

**Table S3** Multivariable logistic regression of predictors of palliative care needs sensitivity - analysis - KCCQ removed from definition of palliative care needs

**Table S4** Multivariable logistic regression of predictors of palliative care needs best fit model using backwards selection

Table S5. Multivariable logistic regression of predictors of palliative care needs best fit model using backwards selection - sensitivity analysis - KCCQ removed from definition of palliative care needs

**Supplementary Figures**

**Figure S1** Calculation of days alive and out of hospital.

**Figure S2** Screening and recruitment.

## **Table S1** **Completion of patient-reported outcome measures**

|  | **Baseline** | **4**  **month** | **8**  **month** | **12 month** | **16 month** | **20 month** | **24 month** |
| --- | --- | --- | --- | --- | --- | --- | --- |
| Study visit not possible due to time in study | 0 | 0 | 2 (1) | 24 (9 | 57 (21) | 88 (32) | 124 (46) |
| Died | 0 | 23 (9) | 48 (18) | 64 (24) | 80 (29) | 91 (34) | 97 (36) |
| Possible study assessments | 272 (100) | 249 (92) | 222 (82) | 184 (68) | 135 (93) | 93 (34) | 51 (19) |
| **Study assessment (% of possible study assessments)** | | | | | | | |
| Completed at least 1 PROM | 272 (100) | 187 (75) | 159 (72) | 136 (74) | 94 (70) | 61 (66) | 34 (67) |
| Did not complete any PROMs | 0 | 4 (2) | 4 (3) | 2 (1) | 2 (2) | 2 (3) | 0 |
| Did not attend for reasons other than death | 0 | 58 (23) | 59 (27) | 46 (25) | 39 (29) | 30 (32) | 17 (33%) |
| **Missing PROM (% of possible assessments)** | | | | | | | |
| HADS-Anxiety | 9 (3) | 72 (29) | 68 (31) | 55 (30) | 50 (37) | 33 (36) | 19 (37) |
| HADS-Depression | 5 (2) | 65 (26) | 66 (30) | 55 (30) | 43 (32) | 32 (34) | 17 (33) |
| KCCQ overall | 0 (0) | 62 (25) | 63 (28) | 48 (26) | 41 (30) | 32 (34) | 17 (33) |
| ESAS overall | 3 (1) | 65 (26) | 64 (29) | 48 (26) | 41 (30) | 32 (34) | 17 (33) |
| SF-12 Physical | 23 (9) | 73 (29) | 75 (34) | 56 (30) | 49 (36) | 36 (38) | 19 (37) |
| SF-12 Mental | 23 (9) | 73 (29) | 75 (34) | 56 (30) | 49 (36) | 36 (38) | 19 (37) |
| Values expressed as n (%)  ESAS= Edmonton symptom assessment scale; HADS= hospital anxiety depression scale; KCCQ= Kansas City Cardiomyopathy Questionnaire; PROM= patient reported outcome measure; SF-12= short form 12. | | | | | | | |

## **Table S2 PROM severity during follow-up**

|  | **Baseline** | **4**  **month** | **8**  **month** | **12 month** | **16 month** | **20 month** | **24 month** |
| --- | --- | --- | --- | --- | --- | --- | --- |
| **Completed at least 1 PROM** | **n=272** | **n= 187** | **n=159** | **n= 136** | **n=94** | **n= 61** | **n= 34** |
| **Severe PROM (% of those with PROM information)** | | | | | | | |
| HADS-Anxiety | 17 (7) | 4 (2) | 4 (3) | 3 (2) | 1 (1) | 1 (2) | 2 (6) |
| HADS-Depression | 10 (4) | 4 (2) | 7 (5) | 2 (2) | 3 (3) | 5 (8) | 2 (6) |
| KCCQ overall | 95 (35) | 28 (15) | 27 (17) | 24 (18) | 14 (15) | 14 (23) | 6 (18) |
| ESAS overall | 26 (10) | 13 (7) | 13 (8) | 13 (10) | 7 (8) | 5 (8) | 5 (15) |
| SF-12 Physical | 35 (14) | 28 (16) | 30 (20) | 17 (13) | 10 (12) | 10 (18) | 6 (19) |
| SF-12 Mental | 13 (5) | 5 (3) | 5 (3) | 5 (4) | 0 | 3 (5) | 1 (3) |
| Overall Severe | 114 (42) | 52 (28) | 55 (35) | 41 (30) | 24 (26) | 22 (36) | 12 (34) |
| **Number of Severe PROMs** | | | | | | | |
| 0 | 158 (58) | 135 (72) | 104 (65) | 95 (70) | 70 (75) | 39 (64) | 22 (66) |
| 1 | 59 (22) | 31 (17) | 36 (23) | 26 (19) | 16 (17) | 13 (21) | 6 (17 |
| 2 | 38 (14) | 15 (8) | 11 (7) | 10 (7) | 4 (4) | 4 (7) | 4 (11) |
| 3 | 10 (4) | 3 (2) | 5 (3) | 2 (2) | 4 (4) | 3 (5) | 0 |
| 4 | 4 (2) | 3 (2) | 2 (1) | 3 (2) | 0 | 2 (3) | 2 (6) |
| 5 | 3 (1) | 0 | 1 (1) | 0 | 0 | 0 | 0 |
| Values expressed as n (%)  ESAS= Edmonton symptom assessment scale; HADS= hospital anxiety depression scale; KCCQ= Kansas City Cardiomyopathy Questionnaire; PROM= patient reported outcome measure; SF-12= short form 12. | | | | | | | |

## **Table S3 Multivariable logistic regression of predictors of palliative care needs - sensitivity analysis - KCCQ removed from definition of palliative care needs**

| **Variable** | **OR** | **95% CI** | **p** |
| --- | --- | --- | --- |
| MAGGIC risk score, per unit increase | 1.39 | (0.96, 2.01) | 0.078 |
| AKPS score, per 10 unit increase | 0.98 | (0.94, 1.01) | 0.91 |
| NAT-PD-HF significant need | 1.12 | (0.44, 2.87) | 0.816 |
| KCCQ summary score < 29 | 3.44 | (1.20, 9.88) | **0.022** |
| HADS Depression summary score, per unit increase | 1.00 | (0.95, 1.14) | 0.954 |
| HADS Anxiety summary score, per unit increase | 1.05 | (0.95, 1.17) | 0.355 |
| ESAS summary score, per unit increase | 1.01 | (0.98, 1.03) | 0.584 |
| SF-12 Physical summary score, per unit increase | 0.97 | (0.92, 1.03) | 0.378 |
| SF-12 Mental summary score, per unit increase | 0.97 | (0.92, 1.03) | 0.300 |
| AKPS= Australia-modified Karnofsky Performance Status scale; CI= confidence interval; ESAS= Edmonton symptom assessment scale; HADS= hospital anxiety and depression scale; KCCQ= Kansas City Cardiomyopathy Questionnaire; MAGGIC = Meta-Analysis Global Group in Chronic Heart Failure; NAT-PD-HF= needs assessment tool progressive disease heart failure. OR= odds ratio; SF-12= short form-12. | | | |

## **Table S4 Multivariable logistic regression of predictors of palliative care needs best fit model using backwards selection**

| **Variable** | **OR** | **95% CI** | **p** |
| --- | --- | --- | --- |
| MAGGIC risk score, per 5 unit increase* | 1.01 | (0.75, 1.36) | 0.946 |
| AKPS score, per 10 unit increase | 0.98 | (0.95, 1.00) | 0.053 |
| KCCQ overall summary score < 29 | 0.96 | (0.93, 0.98) | **< 0.001** |
| SF-12 Mental summary score, per unit increase | 0.97 | (0.94, 1.00) | 0.085 |
| AKPS= Australia-modified Karnofsky Performance Status scale; CI= confidence interval; ESAS= Edmonton symptom assessment scale; HADS= hospital anxiety and depression scale; KCCQ= Kansas City Cardiomyopathy Questionnaire; MAGGIC = Meta-Analysis Global Group in Chronic Heart Failure; NAT-PD-HF= needs assessment tool progressive disease heart failure. OR= odds ratio; SF-12= short form-12.  *MAGGIC variable was forced to be kept in the model during selection process | | | |

## **Table S5 Multivariable logistic regression of predictors of palliative care needs best fit model using backwards selection - sensitivity analysis - KCCQ removed from definition of palliative care needs**

| **Variable** | **OR** | **95% CI** | **p** |
| --- | --- | --- | --- |
| MAGGIC risk score, per 5 unit increase* | 1.40 | (0.98, 2.00) | 0.061 |
| AKPS score, per 10 unit increase | 0.97 | (0.94, 1.00) | 0.034 |
| KCCQ summary score < 29 | 5.43 | (2.23, 13.2) | **< 0.001** |
| SF-12 Mental summary score, per unit increase | 1.10 | (1.01, 1.19) | 0.024 |
| AKPS= Australia-modified Karnofsky Performance Status scale; CI= confidence interval; ESAS= Edmonton symptom assessment scale; HADS= hospital anxiety and depression scale; KCCQ= Kansas City Cardiomyopathy Questionnaire; MAGGIC = Meta-Analysis Global Group in Chronic Heart Failure; NAT-PD-HF= needs assessment tool progressive disease heart failure. OR= odds ratio; SF-12= short form-12.  *MAGGIC variable was forced to be kept in the model during selection process | | | |

## **Figure S1 Calculation of days alive and out of hospital**

**
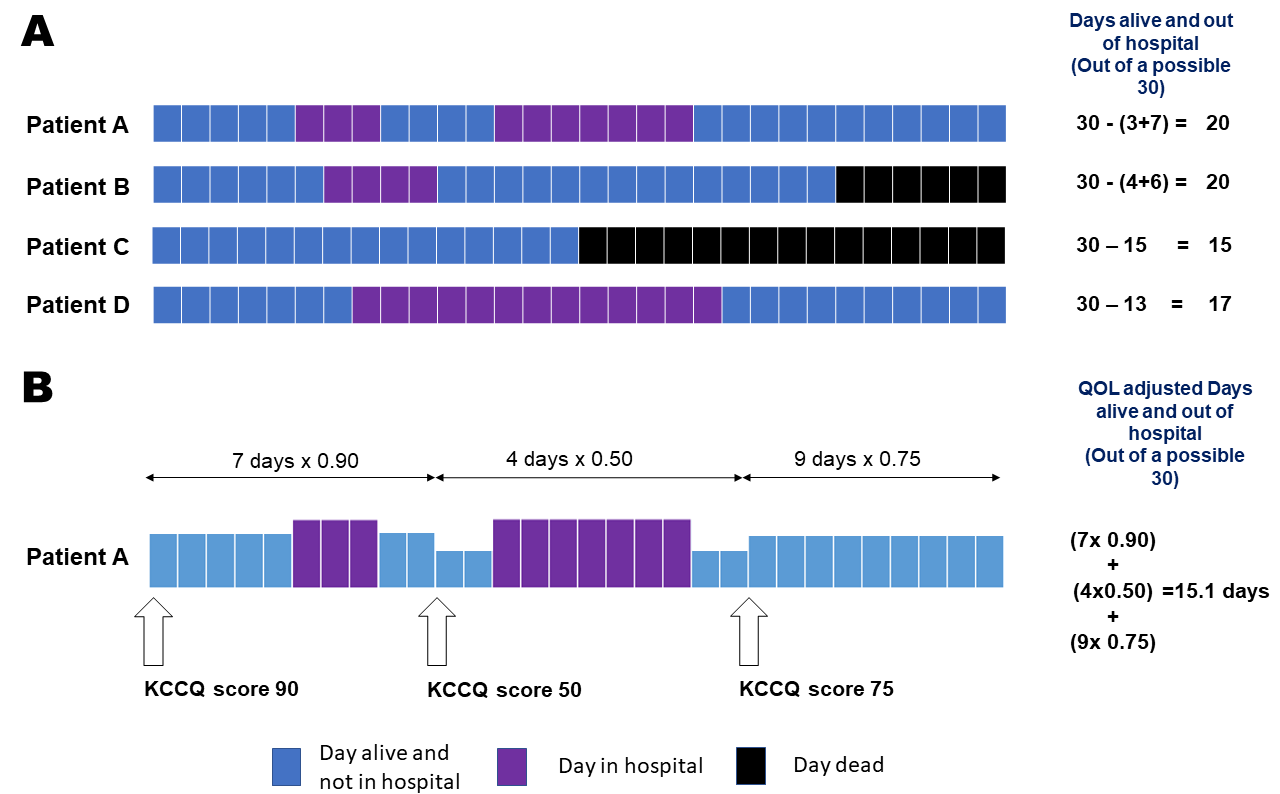
**

KCCQ= Kansas City Cardiomyopathy Questionnaire; QOL= quality of life. **A**= days alive and out of hospital calculation. **B**= Quality of life adjusted days alive and out of hospital calculation.

## **Figure S2 Screening and recruitment**

**
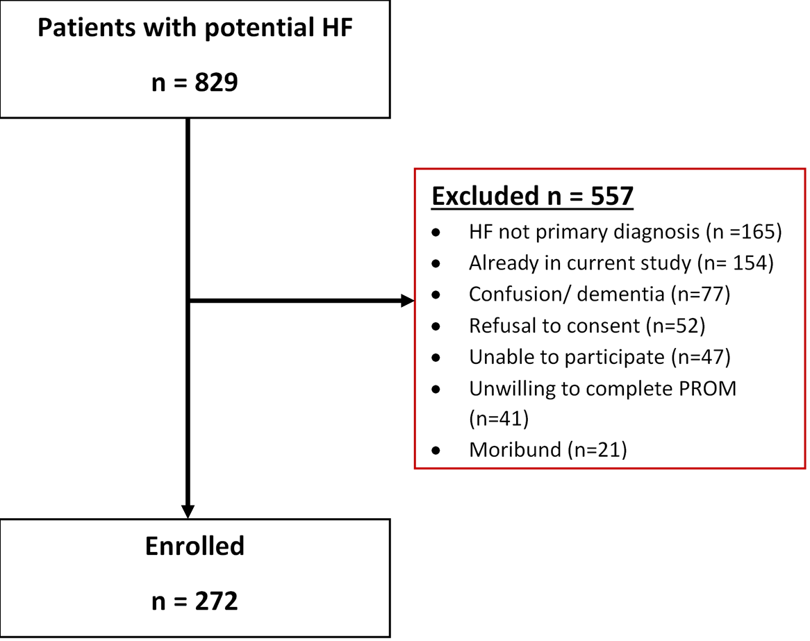
**
